# Supplementary material for: Integrative Analyses Reveal the Anticancer Mechanisms and Sensitivity Markers of the Next-Generation Hypomethylating Agent NTX-301
Source: Cancers (Basel). 2023 Mar 13;15(6):1737. doi: 10.3390/cancers15061737 (PMC10046470; doi:10.3390/cancers15061737)
Supplement: Supplementary file 1 [file cancers-15-01737-s001.zip › cancers-2262970-sup fig.pdf]

Supplementary Figures and Legends

Integrative analyses reveal the anticancer mechanisms and sensitivity markers of the next-generation hypomethylating agent NTX-301

Supplementary Figure S1

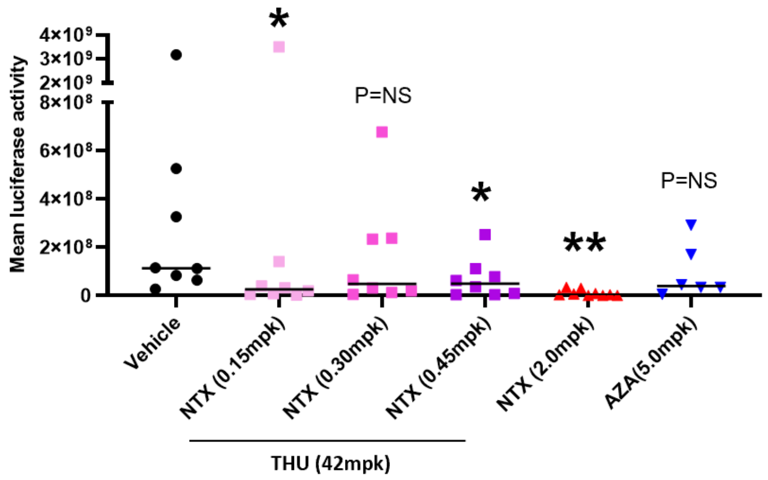

Supplementary Figure S1. Comparison of the efficacies of NTX-301, NTX-301+THU, and AZA in vivo. In NOD/SCID mice transplanted with luciferase-labeled MV4-11 cells (n=8 per group), luciferase activities for tumor growth were measured by the quantification of bioluminescence emission (photons/sec) upon treatment with NTX-301+THU (0.15. 0.30, and 0.45 mg/kg p.o. + 42.0 mg/kg), NTX-301 (2.0 mg/kg p.o.), or AZA (5.0 mg/kg i.p.). **Fig. 3** is an unpublished part of a previous mouse study (Blood Cancer J. 2022 Apr 11;12(4):57. doi: 10.1038/s41408-022-00664-y), and **Supplementary Figure S1** represents a combination of the data shown in **Fig. 3** of this manuscript and data from the previous mouse study to directly compare the treatments. *P*

values (vs. vehicle) are specified and marked as follows: *, p<0.05; **, p<0.001; NS, not significant. AZA, azacitidine; p.o., oral administration; i.p., intraperitoneal administration; THU, tetrahydrouridine.

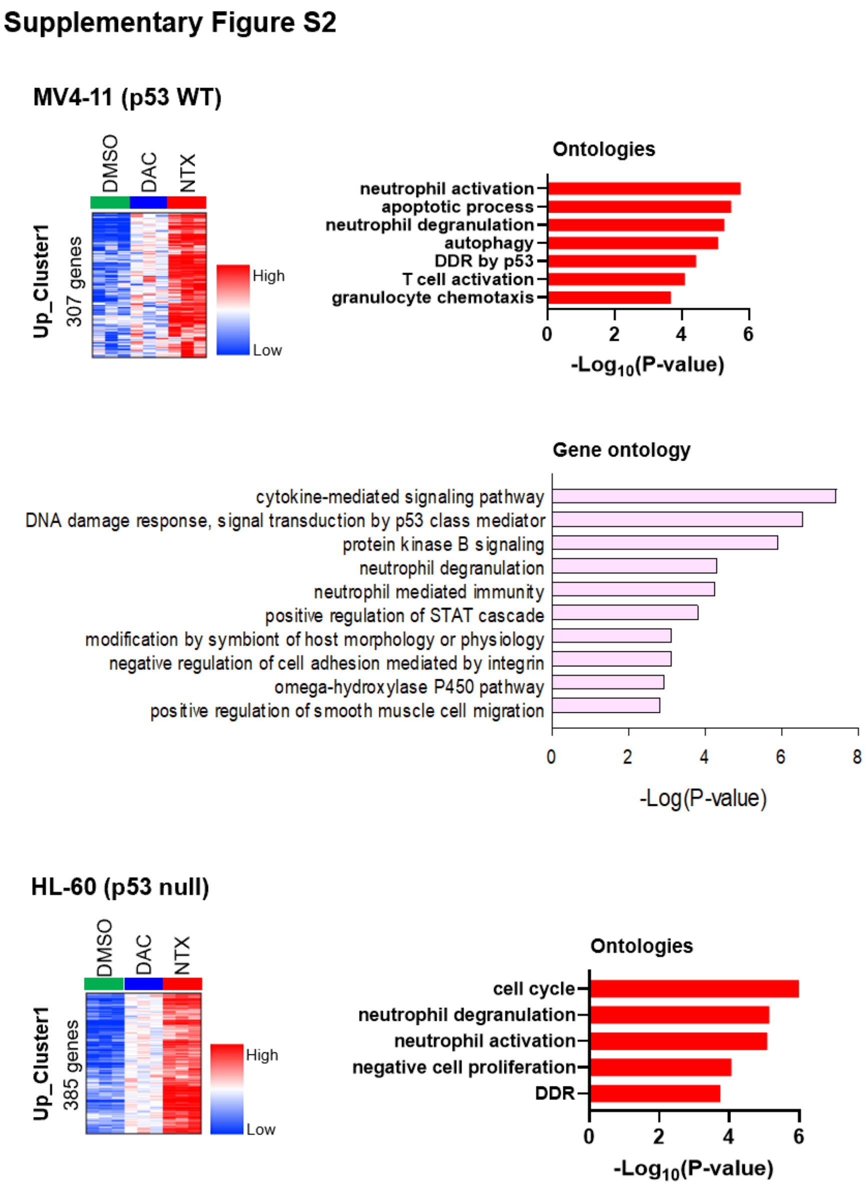

Supplementary Figure S2. Heatmaps showing gene sets that were more strongly upregulated (Up_cluster) by NTX-301 than by DAC in MV4-11 (top) and HL-60

(bottom) cells and bar plots showing the results of Gene Ontology analysis investigating the biological features of each gene set.

Supplementary Figure S3

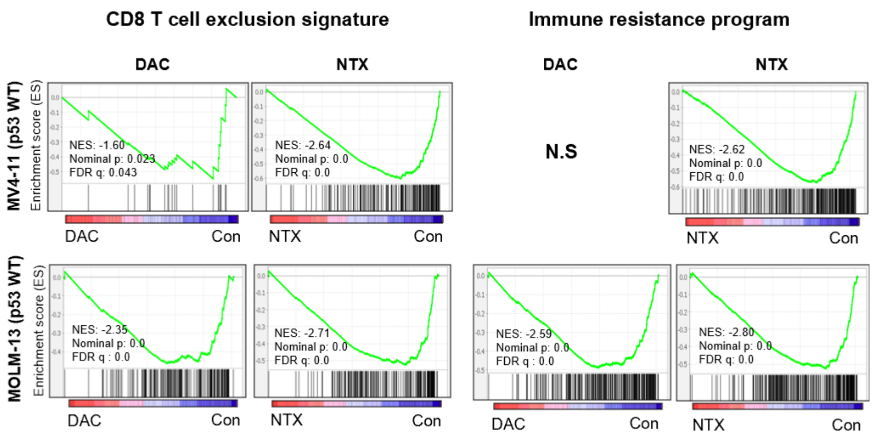

Supplementary Figure S3. Transcriptome analysis revealed NTX-301-induced transcriptional reprogramming in AML cells. GSEA plots show significant enrichment of two signatures, which are associated with immunotherapy resistance (CD8 T cell exclusion signature and immune resistance program), among the transcriptomic changes induced by NTX-301 or DAC in MV4-11 and MOLM-13 cells. A GSEA plot showing statistical insignificance (N.S.) is omitted.

Supplementary Figure S4

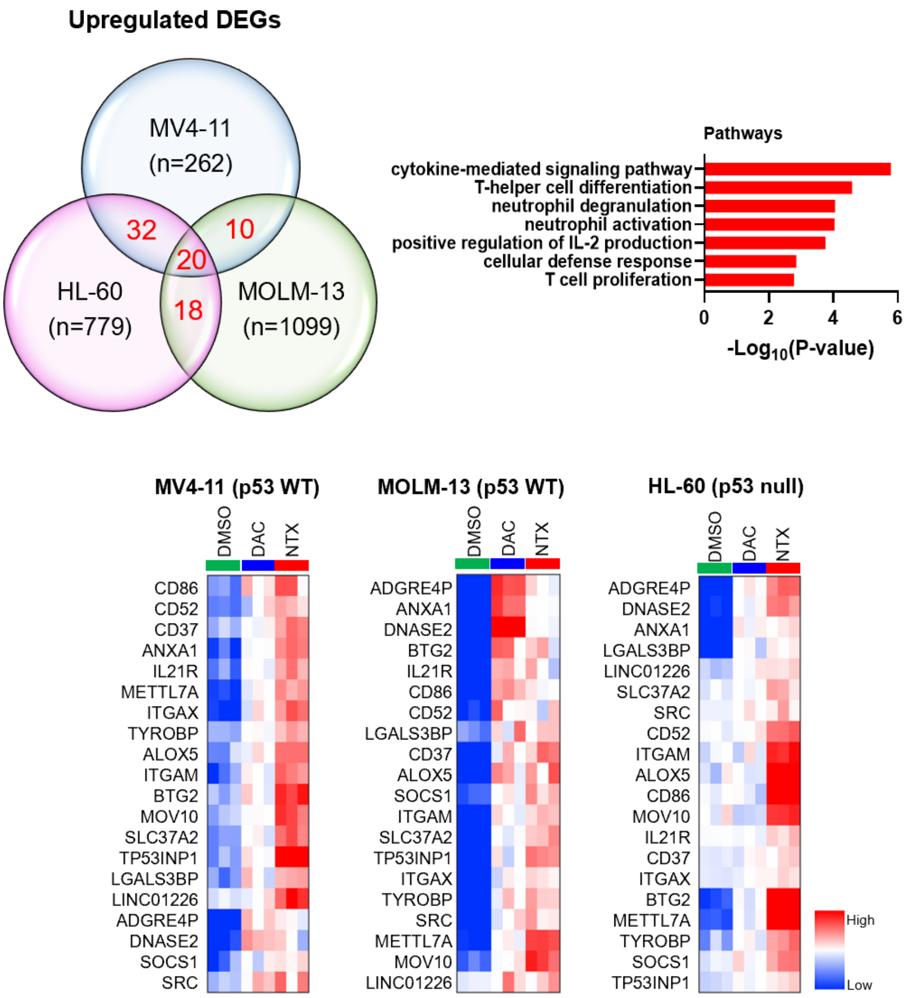

Supplementary Figure S4. Identification of 20 genes that were commonly upregulated by NTX-301 in three AML cell lines, namely, the MV4-11, HL-60, and MOLM-13 cell lines. The Venn diagram and heatmaps show the 20 genes, and the bar plot shows biological pathways with which the genes are significantly associated.

Supplementary Figure S5

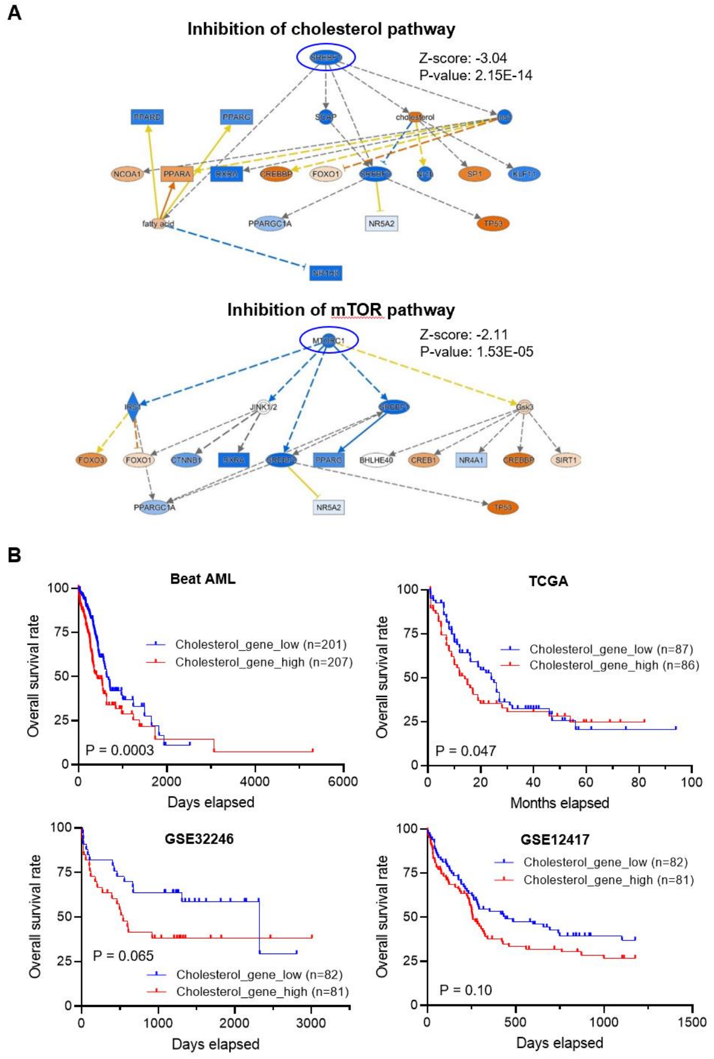

Supplementary Figure S5. Gene set enrichment analysis using genes that were downregulated by NTX-301 revealed the involvement of cholesterol biosynthesis and the mTOR pathway and showed the clinical benefit of these MoAs. **A,** IPA highlighting the inhibition of SREBF1/2 and mTORC1/2, which were predicted to be upstream regulators of the downregulated genes. Z scores and *p* values are shown. **B,** Kaplan–Meier curves showing that the cholesterol biosynthesis-related genes that were downregulated by NTX-301 treatment are significantly associated with the overall survival of AML patients in four cohorts (Beat AML, TCGA, GSE32246, and

GSE12417). The log-rank *P* values are shown.

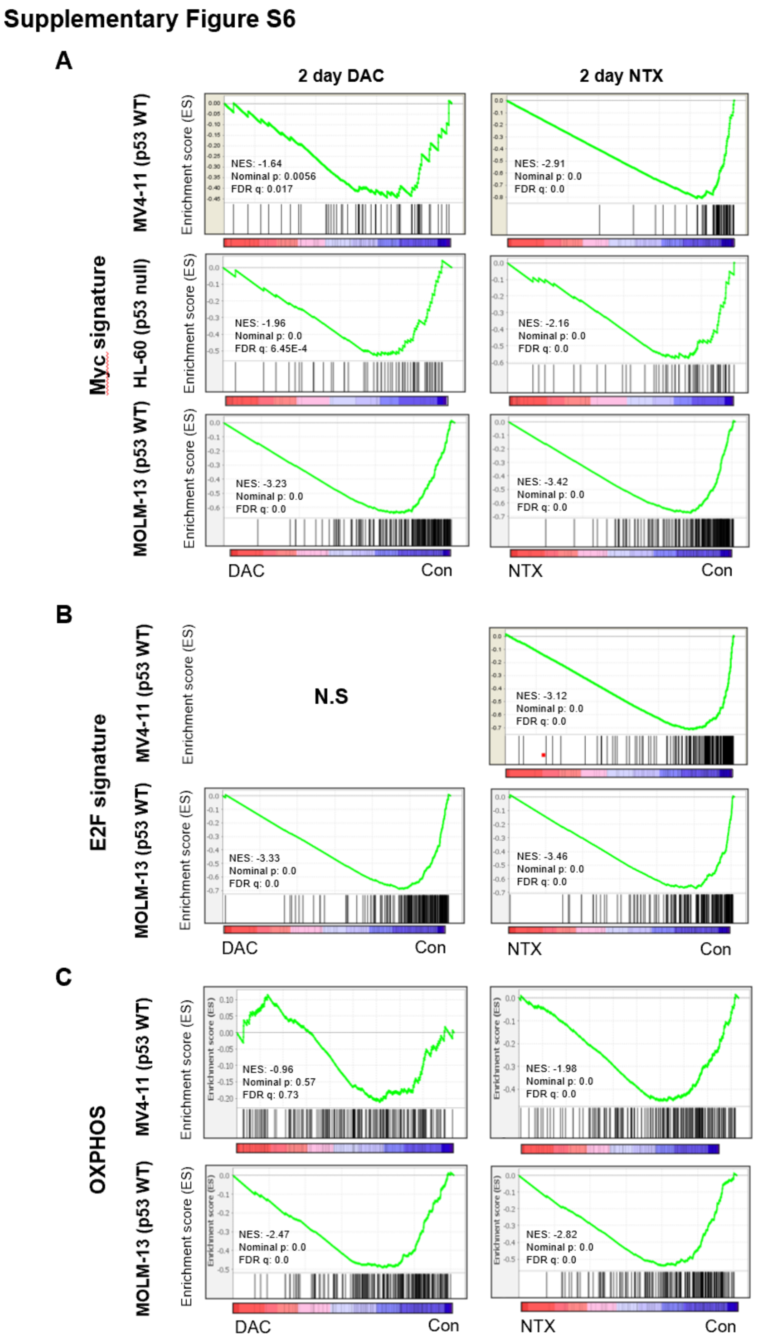

Supplementary Figure S6. Transcriptome analysis reveals that NTX-301 suppresses transcriptional programs that are associated with oncogenic activity in AML. **A-C,** GSEA plots showing significant enrichment of Myc (**A**), E2F (**B**), and oxidative phosphorylation (OXPHOS) signatures (**C**) among the changes in the transcriptome

induced by NTX-301 or DAC in MV4-11, HL-60, and MOLM-13 cells. A GSEA plot showing statistical insignificance (N.S.) is omitted.

Supplementary Figure S7

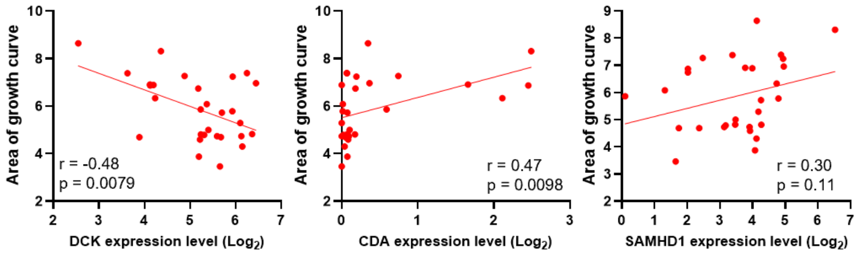

Supplementary Figure S7. Transcriptome data revealed correlations between the efficacy of NTX-301 and the expression levels of genes that are involved in HMA metabolism (*DCK* and *CDA*) and triphosphohydrolase activity (*SAMHD1*). The scatter plots show correlations between the expression levels of *DCK*, *CDA*, and *SAMHD1* and AUC values in blood CCLs after NTX-301 treatment. Correlation coefficients (r) and *p* values are shown.

Supplementary Figure S8

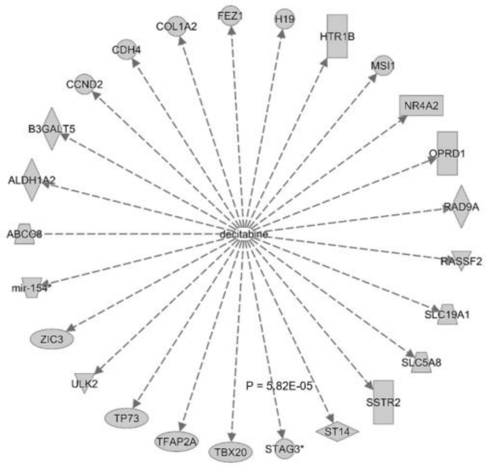

Supplementary Figure S8. IPA shows the significant enrichment of DAC-regulated genes among the downstream genes of DMR352. The *P* value is shown.
